# The Effect of Vibration Mixing on the Mechanical Properties of Steel Fiber Concrete with Different Mix Ratios

**DOI:** 10.3390/ma14133669

**Published:** 2021-06-30

**Authors:** Chunyu Zhang, Yikai Sun, Jianguo Xu, Bo Wang

**Affiliations:** School of Water Conservancy Engineering, Zhengzhou University, Zhengzhou 450001, China; zcy18334930965@gs.zzu.edu.cn (C.Z.); kai20210610@163.com (Y.S.); wangbo@zzu.edu.cn (B.W.)

**Keywords:** steel-fiber-reinforced concrete, vibration stirring, mechanical properties, microstructure

## Abstract

This work addresses how vibration stirring, steel-fiber volume ratio, and matrix strength affect the mechanical properties of steel-fiber-reinforced concrete. The goal of the work is to improve the homogeneity of steel-fiber-reinforced concrete, which is done by comparing the mechanical properties of steel-fiber-reinforced concrete fabricated by ordinary stirring with that fabricated by vibration stirring. The results show that the mechanical properties of steel-fiber-reinforced concrete produced by vibration mixing are better than those produced by ordinary mixing. The general trend is that the mechanical properties of steel-fiber concrete have a linear relationship with the matrix strength and the volume ratio of steel fiber. The best mechanical properties are obtained for a steel-fiber volume ratio of less than 1%. We have also established calculation models for the mechanical performance index of vibration, mixing steel-fiber concrete based on the test results. Microscopic studies show that vibration stirring optimizes the microstructure of the transition zone between the concrete interface and the slurry, and improves the homogeneity of the steel-fiber-reinforced concrete, and enhances the adhesion between the mixture components.

## 1. Introduction

With the continuous improvements in engineering construction levels, concrete has gradually become the most widely used engineering material. Concrete is made of cementitious materials, aggregates and water in an appropriate proportion and is mixed to make a mixture with a certain degree of plasticity. It has a high compressive performance, but its tensile performance is only 1/10–1/20 of its compressive strength. Therefore, improving the ratio of concrete tensile and compressive strength is an important aspect of concrete modification. The incorporation of steel fiber can effectively improve the tensile properties of concrete, enhance cracking control, and improve the overall performance of structural members. Steel-fiber-reinforced concrete is an extremely inhomogeneous composite material prepared and conserved by steel fiber, cement, sand and stone, etc. [1,2,3,4] The performance of steel fiber concrete is closely related to material properties, mix ratio and maintenance conditions [5,6,7,8,9,10]. In terms of material properties, Marcalikova, Z et al. [11] determined the mechanical characteristics of fiber-reinforced concrete with straight and hooked fibers. In terms of mix ratio, Jeong et al. [12] investigated the effect of strength and the aspect ratio of steel fiber on the mechanical properties of high-performance SFRC. Won-Chang et al. [13] studied the effect of steel fiber tensile strength and aspect ratios on the crack performance of high-strength concrete. In terms of maintenance conditions, Yang et al. [14] conducted research on the influence of multiple environmental factors on the compression and bending performance of reinforced concrete columns. Lee [15] studied the influence of concrete strength combined with fiber content in the residual flexural strengths of fiber-reinforced concrete.

The above research shows that adding steel fiber to concrete can effectively improve the mechanical properties of concrete, but there are also obvious limitations: steel fiber is easy to agglomerate during the mixing process, and it is difficult to evenly distribute it in the mixture; the bonding strength between steel fiber and concrete is not enough, which affects the tensile effect. The random distribution of steel fibers in reinforced concrete can effectively hinder the expansion of micro-cracks and the formation of macro-cracks in the concrete, and transfer stress across the crack surface after cracking, significantly improving the brittle behavior of concrete [16,17,18,19]. When the steel fiber concrete is damaged, most of the fibers are pulled out instead of being broken. Therefore, improving the bond strength between the fiber and the matrix is the main controlling factor to improve the fiber reinforcement effect. When the concrete mix ratio is the same, its comprehensive performance depends on the level of homogeneity and bonding strength. Therefore, the mixing process has had increasing attention paid to it in recent years. In the ordinary mixing process, the bonding force between the various components of the mixture makes it difficult to mix the steel fiber concrete evenly, which affects the performance and application of the steel fiber concrete. To address this shortcoming, vibration stirring has been developed in recent years. Vibration stirring involves ordinary forced stirring supplemented by a vibration which can not only improve the uniformity of the mixture, but also reduce the resistance of the mixing process, reduce the energy consumption of the mixing equipment, and improve the mixing efficiency of the mixture [20,21,22,23,24]. Chang’an University invented the vibrating mixer accordingly. The vibrating mixer is used to vibrate the mixture, which accelerates the hydration reaction and increases the speed of the mortar to wrap the aggregate, thereby improving the overall performance of the concrete [25]. Huang et al. [26] studied the effect of vibration mixing on the performance of concrete under different curing conditions and pointed out that vibration mixing has broad prospects in the industrial application of concrete. Zhao et al. [27] and others used the orthogonal test method to determine the reasonable values of mixing time, wet mixing time, vibration frequency and mixing speed, and analyzed the influence of vibration mixing on the compressive strength of cement stabilized crushed stone mixture. Yang et al. [28] optimized the mixing ratio of concrete through vibration mixing technology, verifying the superior role of vibration mixing technology in concrete mixing. The above research mainly analyzes the optimization effect of vibration mixing on the performance of plain concrete and concrete mix ratio, but has not done a systematic study on the influence of vibration mixing technology on reinforced concrete with different mix ratios. Using vibration mixing technology to improve the mixing process of steel fiber concrete is a new concept, a new method and a new technology. At present, there are relatively few studies on the mechanism and influence of vibration mixing on steel fiber concrete. Through systematic experimental research, this paper analyzes the influence of vibration mixing on the mechanical properties of reinforced concrete under different fiber volume ratios and matrix strengths. It is found that vibration mixing can improve the homogeneity and bonding strength of steel fiber concrete, thereby improving the mechanical properties of steel fiber concrete. Applying the research conclusions to actual projects will produce certain economic and social benefits, which is of far-reaching significance for the development of vibration mixing technology in the industrial application of steel fiber concrete.

## 2. Experiment

### 2.1. Raw Materials

**Figure 1:**
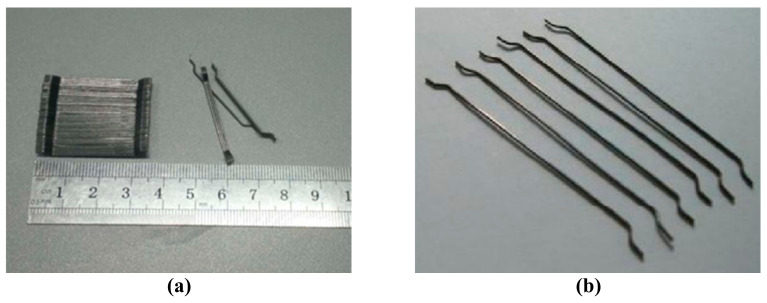
End-hook-type steel fiber. (**a**) Fiber length; (**b**) Fiber shape.

Tianjin Hengfeng (Tianjin, China) end-hook-type steel fiber was used for these experiments; the fibers were 30 mm long with 0.55 mm equivalent diameter, for a length-to-diameter ratio of 64. The ensile strength was 1000 MPa. As shown in Figure 1. The cement was P·O 42.5 ordinary Portland cement, fly ash adopts Class I fly ash. The coarse aggregate is made of crushed stones with a particle size of 5–20 mm, with good particle size distribution and a mud content of less than 1%. The relevant physical properties are shown in Table 1. The fine aggregate is selected from well-graded medium sand, the fineness modulus is controlled at 2.66, the apparent density is 2543 kg/m^3^, and the loose bulk density is 1514.5 kg/m^3^. The sieve classification results of the sand are shown in the following Table 2. The admixture selects polycarboxylic acid series high performance water reducer.

### 2.2. Experimental Method and Mixture Proportion

The tests were conducted following the relevant standards and specifications [29,30]. The mixing method (ordinary stirring and vibration stirring), matrix strength (CF40, CF50, CF60), and steel-fiber volume ratio (0%, 0.5%, 1.0%, 1.5%, and 2.0%) are the experimental parameters, and the mechanical property index is the object of investigation. The specific calculation process is as follows:

(1) Determine the water-binder ratio W/B

(1) $$W/B=\frac{\alpha_{a}f_{ce}}{f_{cu.0}+\alpha_{a}\alpha_{b}f_{ce}}$$

In the formula: $\alpha_{a}\alpha_{b}$ is the regression coefficient, which is taken according to relevant specifications; $f_{ce}$ is the 28-day measured compressive strength of cement (MPa); $f_{cu.0}$ is the concrete compounded strength (MPa).

(2) Determine unit water consumption $m_{w0}$

The water-binder ratio is constant. The main factor that affects the workability of the mixture is the water consumption per unit volume. According to the workability requirements and the water reducing efficiency of the water reducer.

(3) Determine the unit amount of cement $m_{c0}$ and fly ash $m_{a0}$

The amount of fly ash in this test is 10% of the mass of the cementitious material. According to the unit water consumption and the water-binder ratio, $m_{c0}$ and $m_{a0}$ are obtained.

(4) Determine the sand rate $\beta_{s}$

Sand rate affects the strength and homogeneity of steel fiber concrete. Taking into account these two aspects, according to relevant specifications and experience, the sand rate is determined by the water-binder ratio and the maximum particle size of the aggregate [31].

According to the above calculation process, the coordination ratio of this test is determined as shown in Table 3.

### 2.3. Experiment Instruments

(1) Vibration mixer

The experiments used a DT60 ZBW (Detong, Shanghai, China) double-horizontal-shaft vibration mixer to fabricate the test pieces [32]. This instrument consists essentially of the stirring drive mechanism, a vibration drive mechanism, and a chain transmission module. The stirring drive mechanism transfers torque through the chain transmission to rotate the stirring shaft, the stirring arm, and the stirring blade, which forces the materials to circulate in the stirring drum. At the same time, the vibration driving mechanism rotates the eccentric vibration shaft at high speed, so that the stirring shaft, the stirring arm, and the stirring blade mounted on the eccentric vibration shaft all vibrate while stirring, which is the essence of vibration stirring. The control variable of the experiments is the vibration factor. Ordinary stirring used the mandatory stirring program. The main performance parameters of the DT60 ZBW instrument are given in Table 4.

(2) Press machines

**Figure 2:**
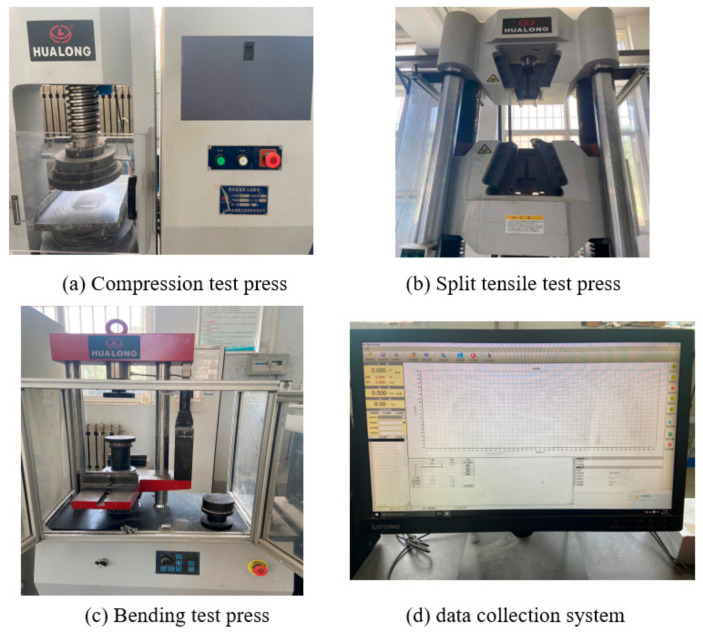
Press and data acquisition system: (**a**) compression test press; (**b**) split tensile test press; (**c**) bending test press; (**d**) data collection system.

As shown in Figure 2, the compressive strength test adopts the WHY-300 microcomputer controlled pressure testing machine of Shanghai Hualong Company (Shanghai, China). The maximum conforms to 300 KN, and the control accuracy is better than 1%. The tensile strength test adopts a ENUO-TEST liquid crystal display electronic universal testing machine. The tensile space is 600 mm, the maximum load is 20 KN, and the measuring range is 0.4–100% KN. It has the characteristics of a high speed regulation accuracy and a stable performance. The flexural strength test adopts the microcomputer YAW 300B flexural testing machine, the maximum test force is 300 KN, the maximum stroke of the piston is 80 mm, and the loading speed error is 5%. The above instruments meet the specific requirements in “General Technical Requirements for Testing Machines” (GB/T2611) [33]. The data acquisition system is MAX-TEST software. The software can automatically obtain the test data results, support the statistical calculation of test data, and the characteristic test data can be marked and displayed on the graph.

(3) Scanning electron microscope

**Figure 3:**
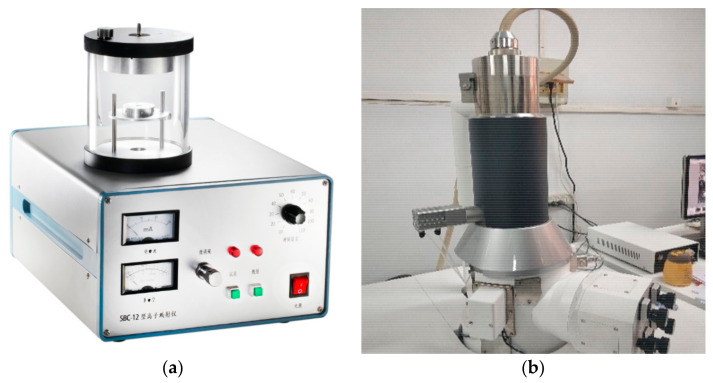
Ion sputtering instrument (**a**) and scanning electron microscope (**b**).

Using SBC-12 ion sputtering instrument (Minyi, Shanghai, China) and KYKY-EM6200 scanning electron microscope (Zhongke, Beijing, China), as shown in Figure 3. The resolution of the instrument is 4.5 nm (30 KV), the magnification is 15×–250,000×, and the sample stage can be adjusted manually.

## 3. Experimental Results and Discussion

By comparing the performance of reinforced concrete with different matrix strengths and different mix ratios under ordinary mixing and vibration mixing, it can be seen that vibration mixing has a significant impact on the performance and failure mode of reinforced concrete. It has different influence rules on the compressive strength, tensile strength, elastic modulus and flexural strength of reinforced concrete.

Table 5 compares the measured mechanical properties for steel-fiber-reinforced concrete produced by ordinary mixing (OM) and vibration mixing (VM).

### 3.1. Research on the Compressive Strength Performance of Concrete Cubes

#### 3.1.1. Variation in Cube Compressive Strength

**Figure 4:**
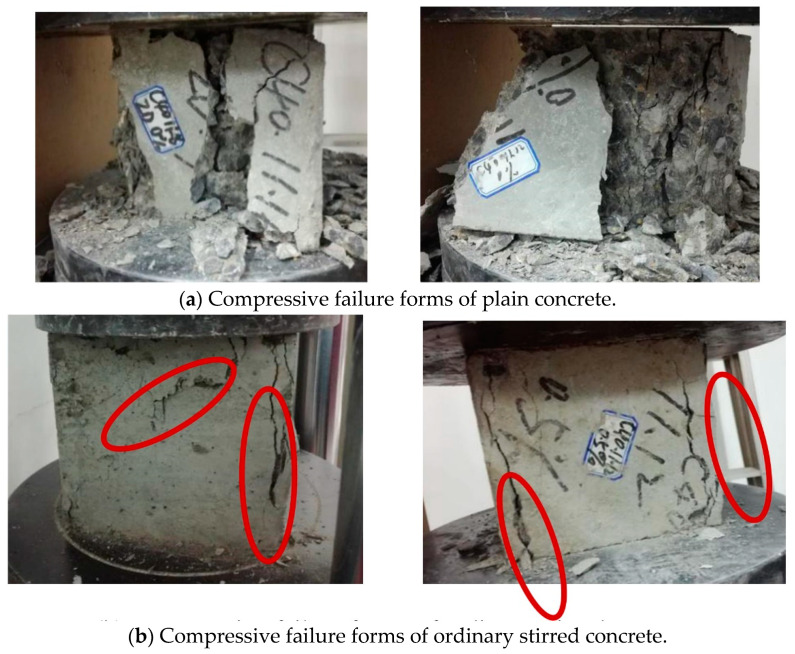

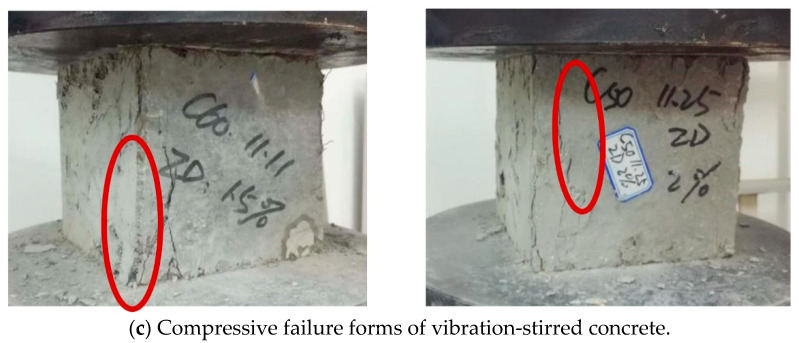
Concrete compressive failure forms: compressive failure forms of plan concrete (**a**), compressive forms of ordinary stirred concrete (**b**), and compressive failure forms of vibration-stirred concrete (**c**).

Figure 4 shows the compressive-failure forms of plain concrete, ordinary stirred concrete, and vibration-stirred concrete, respectively. For specimens without steel fiber, vertical compression deformation and lateral elongation deformation occur first during the compression process, and when the load continues, the cracks develop and expand to the inside of the specimen. The surface concrete began to fall off, and finally accompanied by a brittle sound, the specimen was a wedge-shaped failure. The failure mainly occurred in the transition zone between the cement mortar and the aggregate, and the aggregate itself was not crushed. The cracks of the ordinary mixed steel fiber concrete specimens appeared later, and the cracks increased and developed gradually in the later period. There was less peeling on the surface of the specimens. After the loading was completed, the specimens showed obvious plastic failure. The fracture process of the steel fiber concrete specimens subjected to vibration mixing is roughly similar to that of ordinary mixing, but the amount of peeling on the surface of the specimens is less, the number of cracks is small, the width is narrow, cracking but not scattered, no large through cracks, and it can better maintain the integrity of the test piece. The presentation of different failure forms of the specimens shows that vibration stirring is more conducive to the ability of steel fiber to control the generation and development of cracks, thereby further improving the compressive performance of concrete and avoiding brittle failure.

**Figure 5:**
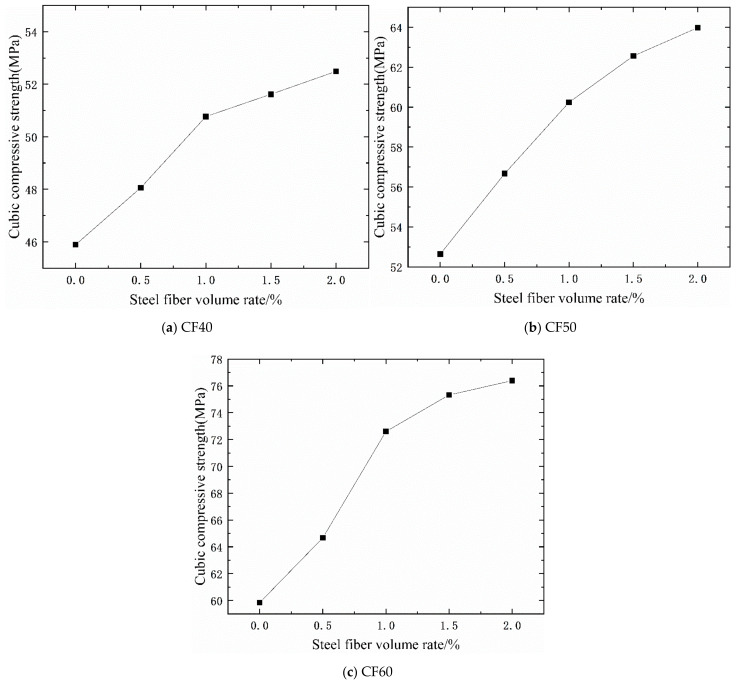
The relationship between cubic compressive strength and steel fiber content. CF40 (**a**), CF50 (**b**), and CF60 (**c**).

It can be seen from Table 5 and Figure 5 that adding steel fiber can increase the cubic compressive strength of concrete. Under the ordinary mixing method, with the increase in the volume ratio of steel fiber, the compressive strength of the cube is continuously improved, and the efficiency of improvement first increases and then decreases. When the concrete grade is CF40, the compressive strength of concrete with a 0.50% steel fiber volume ratio is increased by 4.72% more than that of plain concrete, and the compressive strength of concrete with 1.0% steel fiber volume ratio is 10.63% higher than that of plain concrete, and 1.5% steel fiber volume ratio is increased by 13.79% than that of plain concrete, and the compressive strength of concrete mixed with 2.0% steel fiber volume ratio is 14.38% higher than that of plain concrete. Among them, the addition of steel fiber in the early stage improves the compressive strength of concrete more obviously, and the concrete compressive strength improvement efficiency of 1% steel fiber is the highest, reaching 5.42%. As the volume rate of steel fiber increases in the later period, the lifting efficiency decreases. When the concrete grade is CF50, the compressive strength of concrete mixed with a 2.0% steel fiber volume ratio is increased by 21.37% compared with that of plain concrete. Among them, 0.5% steel fiber added concrete has the highest compressive strength improvement efficiency, reaching 8.06%. When the concrete grade is CF60, the compressive strength of concrete mixed with a 2.0% steel fiber volume ratio is 27.66%, which is higher than that of plain concrete. Among them, the concrete compressive strength improvement efficiency with 1% steel fiber content is the highest, reaching 15.86%.

**Figure 6:**
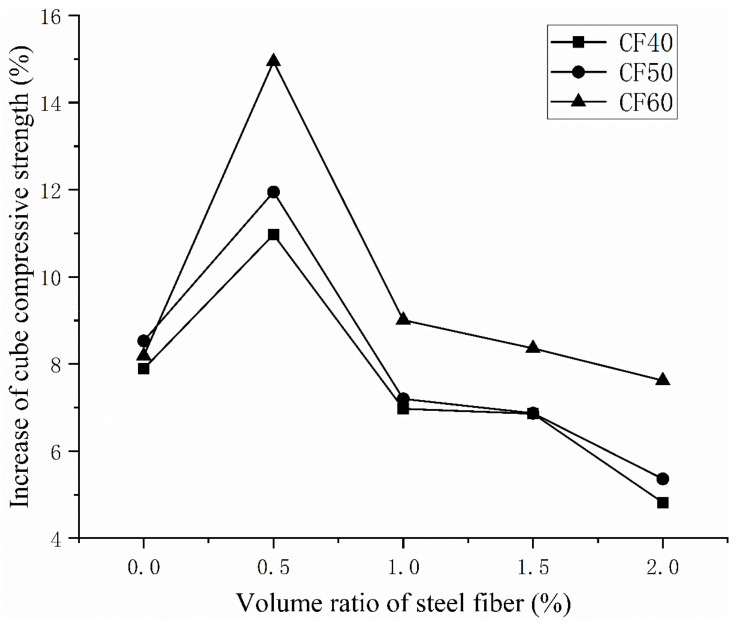
The improvement of the cubic compressive strength of vibration mixing compared with ordinary mixing.

It can be seen from Table 5 that under the vibration mixing mode, as the volume ratio of steel fiber increases, the compressive strength of the cube continues to increase. When the concrete grade is CF40, the compressive strength of concrete mixed with a 2.0% steel fiber volume ratio is 14.1% higher than that of plain concrete. When the concrete grade is CF50, the compressive strength of concrete mixed with a 2.0% steel fiber volume ratio is 18% higher than that of plain concrete. When the concrete grade is CF60, the strength of concrete mixed with a 2.0% steel fiber volume ratio is 26.98% higher than that of plain concrete. Among them, the addition of steel fiber in the early stage improves the compressive strength of concrete more obviously, and the concrete strength improvement efficiency of 0.5% steel fiber is the highest, reaching 19.18%. Figure 6 shows the degree of improvement in the compressive strength of vibrating mixing concrete relative to ordinary mixing methods under different steel fiber volume ratios. It can be seen from the figure that when the amount of steel fiber is 0.5%, the efficiency of vibration stirring is the highest compared with ordinary stirring. Compared with the base concrete, the compressive strength of the CF60 of vibration mixing is increased by 37.38%, which is 14.93% higher than the compressive strength of the ordinary mixing method.

It can be seen from the above test results that when the amount of steel fiber is small, the influence of steel fiber on the compressive strength of concrete is less than the influence of vibration mixing on the compressive strength of concrete. This is because the steel fiber mainly inhibits the expansion of concrete cracks through the viscous stress between the two sides of the concrete cracks and improves the overall performance of the concrete. At this time, the effect of vibration mixing on the workability of concrete is better than the effect of the steel fiber volume rate on compressive strength.

#### 3.1.2. Calculation of Cube Compressive Strength for Vibration-Stirred Concrete

The tests show that the factors affecting the compressive strength of steel-fiber concrete cubes include vibration stirring, matrix strength, and steel-fiber volume ratio. We use the following simple model to calculate the relationship between vibration mixing and the compressive strength of steel-fiber-reinforced concrete cubes:

(2) $$f_{fcu}=f_{cu}\alpha {\left(\frac{W}{B}\right)}^{m}\left(1+bl_{f}\right)$$

(3) $$l_{f}=\frac{r_{f}l_{f}}{d_{f}}$$

where $f_{fcu}$ is the compressive strength (MPa) of the vibration-stirred cube, $f_{cu}$ is the compressive strength (MPa) of the cube of matrix concrete, and α, m, b are the cube compressive strength coefficients of vibration stirring, matrix strength, and steel fiber volume ratio, respectively, W/B is the water-binder ratio for steel-fiber concrete, $l_{f}$ is the characteristic steel-fiber content, $r_{f}$ is the steel-fiber volume ratio, H is the length of steel fiber (mm), and $l_{f}$ is the steel-fiber diameter (mm).

The data fitting analysis gives the cube compressive strength coefficients for vibration stirring, matrix strength, and a steel-fiber volume ratio of 1.06, −0.1, and 0.11, respectively. The cube compressive strength for vibration stirring is calculated as follows:

(4) $$f_{fcu}=1.06f_{cu}{\left(\frac{W}{B}\right)}^{-0.1}\left(1+0.11l_{f}\right).$$

Table 6 compares the theoretical and experimental cube compressive strengths. The error range between the experimental and theoretical results is within 8.43%, the mean ratio is 1.0004, and the mean square error is 0.0361.

### 3.2. Research on Performance of Concrete Elastic Modulus

#### 3.2.1. Variation in Elastic Modulus

**Figure 7:**
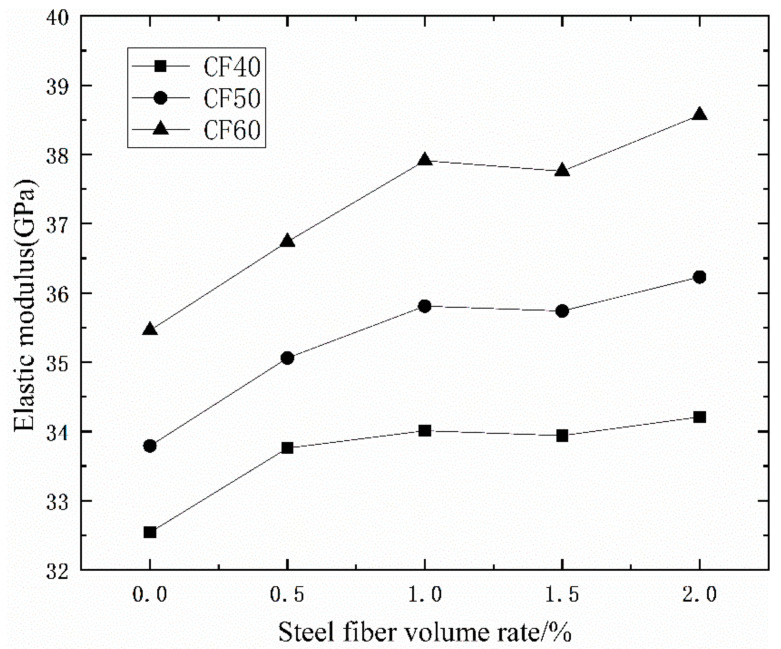
The relationship between the elastic modulus of concrete and steel fiber content.

**Figure 8:**
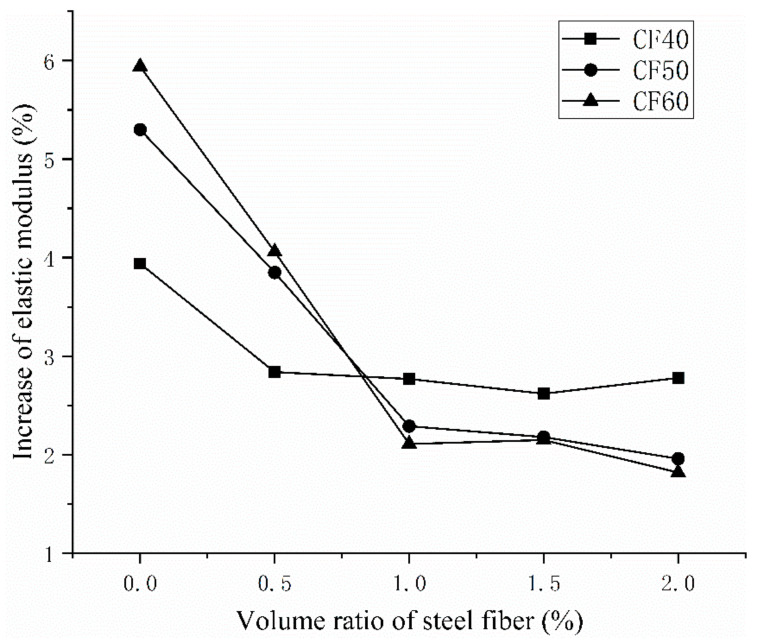
The improvement of the elastic modulus of vibrating mixing concrete compared with ordinary mixing.

Table 5 and Figure 7 and Figure 8 show that, for a given matrix strength, the elastic modulus under vibration stirring and ordinary stirring increases, decreases, and then increases again with the increasing steel-fiber volume ratio. It can be seen from Figure 5 that under the ordinary mixing method, when the volume ratio of steel fiber is 2%, the elastic modulus of concrete increases the most. When the concrete grades are CF40, CF50, and CF60, the elastic modulus of concrete mixed with a 2.0% steel fiber volume ratio increased by 5.13%, 7.22% and 8.77%. It can be seen from Figure 6 that under the same volume ratio of steel fiber, the elastic modulus of concrete after vibration mixing is higher than that of concrete under ordinary mixing methods. As with the concrete grade of CF40, CF50 and CF60, the elastic modulus of plain concrete under the vibration mixing method is 3.93%, 5.30%, and 5.95% higher than that under the ordinary mixing method. With the increase of the volume ratio of steel fiber, the increase in the elastic modulus of concrete under the vibration mixing method gradually decreases compared with that under the ordinary mixing method. When the volume ratio of steel fiber is 2%, the elastic modulus of vibration mixing CF60 is only 1.81% higher than that of ordinary mixed concrete. It can be seen that the effect of vibration mixing on the elastic modulus of plain concrete is stronger than that on the steel fiber concrete. The effect of vibration mixing on the compressive strength of concrete cubes is higher than that on the elastic modulus of concrete.

It can be seen from the test results that when the volume ratio of steel fiber is higher than 1%, the higher the strength of the matrix, the lower the increase in elastic modulus by vibration stirring. The main reason is that when the strength of the matrix increases, the water-binder ratio of concrete decreases and the amount of cementing material is large, and the fine cemented particles cannot be dispersed by vibrating stirring under the condition of vibration pressure of 0.55 MPa.

#### 3.2.2. Calculation of Elastic Modulus of Vibration-Stirred Concrete

Based on the calculation of elastic modulus of ordinary-stirred steel-fiber-reinforced concrete, the calculation of the elastic modulus of vibration-stirred steel-fiber-reinforced concrete is

(5) $$E_{fc}=\frac{10^{5}}{2.2+\frac{34.7}{f_{cu}}}\alpha_{c}\left(1+b_{c}l_{f}\right),$$

where $E_{fc}$ is the elastic modulus of the steel-fiber-reinforced concrete (MPa), and $\alpha_{c}$ and $b_{c}$ are the elastic-modulus coefficients for vibration mixing and for the steel-fiber volume ratio, respectively.

According to the data, the elastic-modulus coefficient is 1.03 and 0.16 for vibration stirring and the steel-fiber volume ratio, respectively. The elastic modulus for vibration-stirred steel-fiber concrete is thus

(6) $$E_{fc}=\frac{1.03\times 10^{5}}{2.2+\frac{34.7}{f_{cu}}}\left(1+0.16l_{f}\right).$$

The error range between experimental and calculated results is within 10%, the mean value of the ratio is 1.0416, and the mean square error is 0.0648.

### 3.3. Research on Splitting Tensile Strength of Concrete

#### 3.3.1. Variation in Splitting Tensile Strength

**Figure 9:**
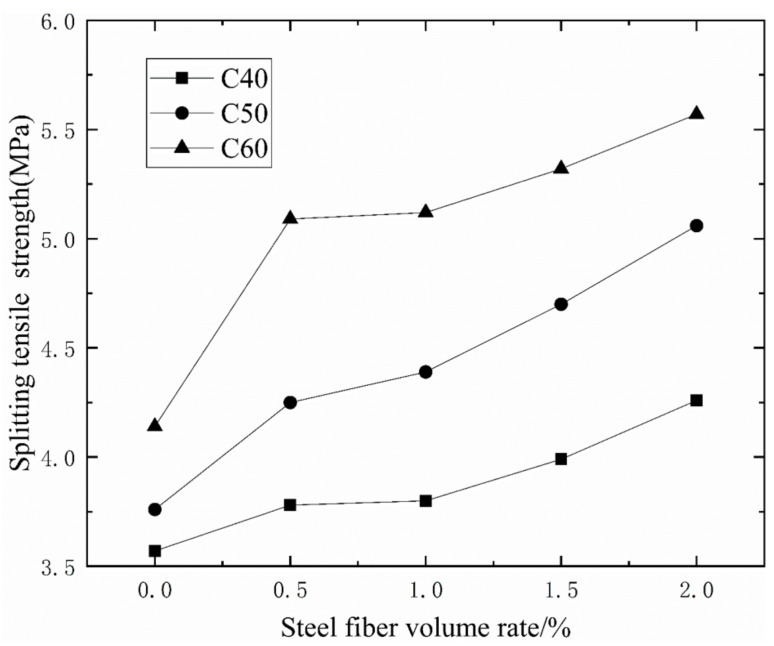
The relationship between the splitting tensile strength of concrete and steel fiber content.

It can be seen from Table 5 and Figure 9 that with the increase of the volume ratio of steel fiber, the splitting tensile strength of concrete continues to increase, and the overall trend is increasing. Under the ordinary mixing method, when the concrete grades is CF40, the fiber content of 0.5%, 1%, 1.5%, and 2%, the splitting tensile strength of concrete increases by 5.88%, 6.44%, 11.76%, and 19.33%. When the concrete grades is CF50, the fiber content of 0.5%, 1%, 1.5%, and 2%, the splitting tensile strength of concrete increases by 13.03%, 16.76%, 25%, and 34.57%. When the concrete grades is CF60, the fiber content of 0.5%, 1%, 1.5%, and 2%, the splitting tensile strength of concrete increases by 22.95%, 23.67%, 28.50%, and 34.54%. Comparing the splitting tensile strength of CF40, CF50, and CF60, it can be seen that the addition of steel fiber in the early stage can significantly increase the splitting tensile strength of concrete. The 0.5% steel fiber content has the highest increase efficiency of concrete strength, up to 22.95%.

**Figure 10:**
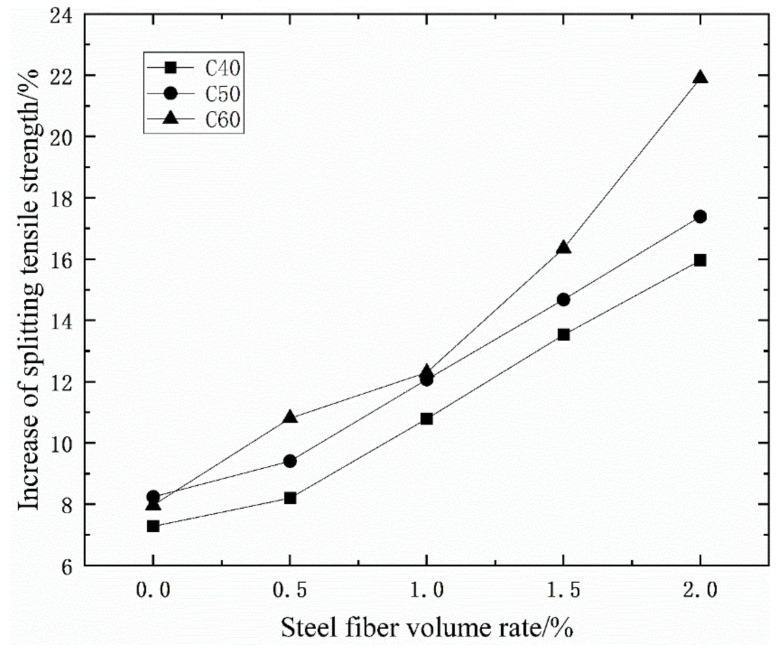
The improvement of the splitting tensile strength of vibrating mixing compared with ordinary mixing.

It can be seen from Table 5 and Figure 10 that when the volume ratio of the steel fiber is the same, the increase in the splitting tensile strength of the vibration stirring is higher than that of the ordinary mixing method. The splitting tensile strength of CF60 with vibration mixing is up to 51.90% higher than the splitting tensile strength of plain concrete. As the volume ratio of steel fiber increases, the splitting tensile strength of vibrating-mixed concrete gradually increases compared to that of ordinary mixed concrete. The splitting tensile strength of vibration stirring CF60 mixed with 2.0% steel fiber volume ratio is 21.90% higher than that of ordinary stirring CF60. In summary, the effect of vibration mixing on the splitting tensile strength of concrete is higher than the effect of vibration mixing on the cubic compressive strength of concrete and the elastic modulus of concrete.

Because the surface of the aggregate will adhere to dust, a water film will form to isolate the cement particles from the aggregate after encountering water, which affects the cementing material and the bond between the steel fiber and the aggregate, thereby affecting the overall strength of the concrete. The high-frequency vibration of vibratory stirring makes the dust attached to the surface of the aggregate peel off and the aggregate is wetted, which accelerates the hydration reaction of cement particles, reduces the generation of steel fiber clusters, and improves the overall strength of the concrete.

#### 3.3.2. Calculation of Splitting Tensile Strength of Vibration-Stirred Concrete

The experiments show that the splitting tensile strength depends only slightly on the matrix strength. Therefore, we consider only the other two factors in the following calculation of the splitting tensile strength of vibration-stirred steel-fiber-reinforced concrete:

(7) $$f_{ft}=f_{t}a_{t}\left(1+b_{t}l_{f}\right),$$

where $f_{ft}$ is the splitting tensile strength of vibration-stirred concrete (MPa), $f_{t}$ is the splitting tensile strength of the matrix concrete (MPa), and $a_{t},\;b_{t}$ are the splitting tensile strength coefficients for vibration stirring and steel-fiber volume ratio, respectively.

Based on the data analysis, the splitting tensile strength coefficients are 1.09 and 0.33 for vibration stirring and steel-fiber volume ratio, respectively. In other words, the splitting tensile strength of vibration-stirred steel-fiber-reinforced concrete may be calculated by using

(8) $$f_{ft}=1.09f_{t}\left(1+0.33l_{f}\right).$$

The error range between experimental and calculated split tensile strength is less than 10%, the average value of the ratio is 0.9892, and the mean square error is 0.0698.

### 3.4. Research on the Flexural Strength of Concrete

#### 3.4.1. Variation in Flexural Strength

**Figure 11:**
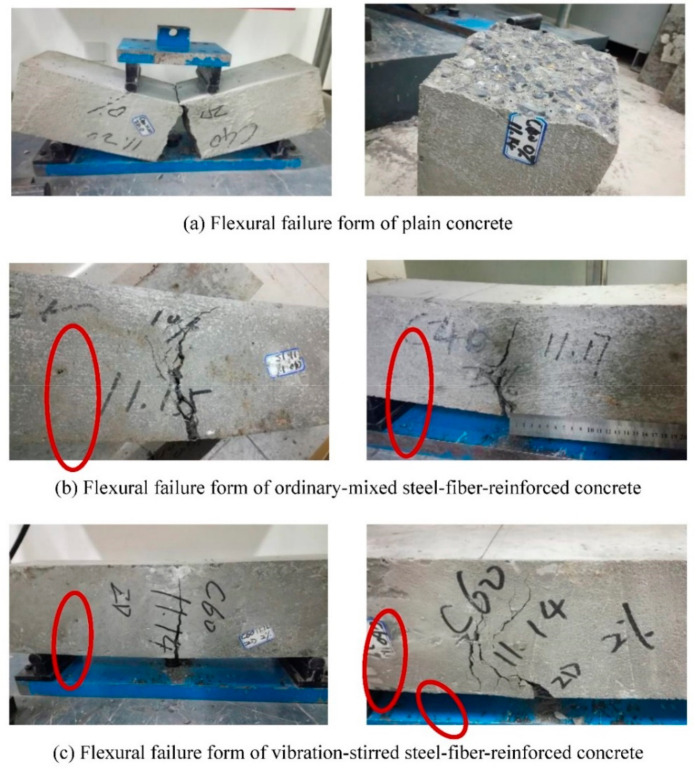
Concrete flexural failure forms. Flexural failure form of plain (**a**), Flexural failure form of ordinary-mixed steel-fiber-reinforced concrete (**b**), and Flexural failure form of vibration-stirred steel-fiber-reinforced concrete (**c**).

Figure 11 shows the forms of flexural failure of plain concrete, ordinary mixed steel fiber concrete and vibration-stirred steel fiber concrete. Vertical cracks appeared at the bottom of the concrete specimen without steel fiber and continued to extend upward. Finally, the specimen broke into two halves from the middle, and the specimen had almost no ductility. Under the load of the ordinary mixed steel fiber concrete specimen, a main crack appeared under the tension zone. Then, some small vertical cracks appeared around the main crack and developed upward, some of the steel fibers were pulled out, and the specimen cracked but was not disconnected. It can be found that the higher the volume ratio of steel fiber, and the greater the ductility of steel fiber concrete under bending failure, the fewer the number of bending cracks, and the narrower the width. The damage shape of the vibration mixing steel fiber concrete specimen is similar to that of ordinary mixing, but the ultimate load is larger when the vibration mixing specimen is destroyed, and the crack shape is also approximate to a straight line, and the position is closer to the center of the specimen. It can be seen that the vibratory stirring makes the material uniformly distributed under the action of high-frequency vibration, and the powder material better fills the voids of the aggregate, which improves the uniformity and integrity of the steel fiber concrete.

**Figure 12:**
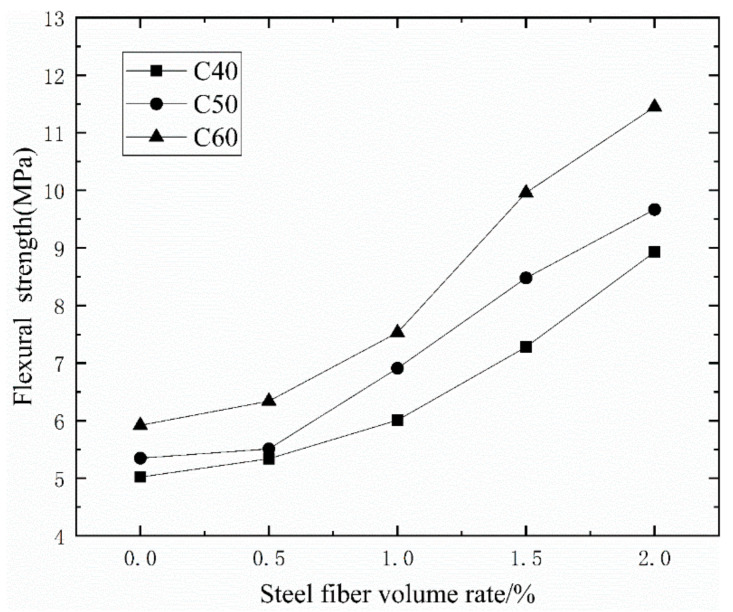
The relationship between the flexural strength of concrete and steel fiber content.

**Figure 13:**
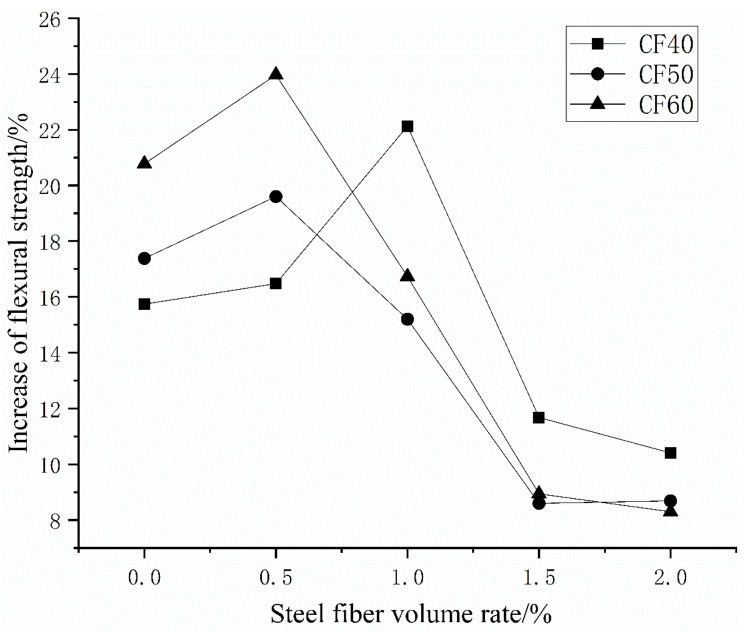
The improvement of the flexural strength of vibrating mixing compared with ordinary mixing.

Table 5 and Figure 12 show that, for a given matrix strength, as the volume ratio of steel fiber increases, the flexural strength of concrete continues to increase. Under ordinary mixing, when the amount of steel fiber is 2%, the flexural strength of CF40 increases by 77.89%, the flexural strength of CF50 increased by 80.75%, and the flexural strength of CF60 increased by 93.41%. Among them, the addition of steel fiber in the early stage does not significantly improve the flexural strength of concrete. When the amount of steel fiber is 1.5%, the efficiency of improving the flexural strength of concrete is the highest. It can be seen from Table 5 and Figure 13 that compared with ordinary stirring methods, the lifting effect of vibration stirring increases first and then decreases with the increase in the amount of steel fiber incorporated. When vibrating stirring CF40 is mixed with 1% steel fiber, the flexural strength is up to 22.13% higher than ordinary stirring. When vibrating stirring, CF50 is mixed with 0.5% steel fiber, the flexural strength is up to 19.60% higher than ordinary stirring. The flexural strength of vibration stirring CF60 when 0.5% steel fiber is added is up to 23.97% higher than that of ordinary stirring.

#### 3.4.2. Calculation of Flexural Strength of Vibration-Stirred Concrete

Experiments show that the flexural strength of steel-fiber-reinforced concrete is affected by various factors, including vibration mixing, matrix strength, and the steel-fiber volume ratio. Therefore, the flexural strength of the steel-fiber-reinforced concrete is calculated as

(9) $$f_{ftm}=f_{tm}a_{tm}{\left(\frac{W}{B}\right)}^{m_{tm}}\left(1+b_{tm}l_{f}\right),$$

where $f_{ftm}$ is the flexural strength of vibration-stirred concrete (MPa), $f_{tm}$ is the flexural strength of the matrix concrete (MPa), and $a_{tm},\;m_{tm},\;b_{tm}$ are the flexural-strength coefficients for vibration stirring, matrix strength, and steel-fiber volume ratio, respectively.

Fitting the data gives the flexural-strength coefficients for vibration stirring, matrix strength, and a steel-fiber volume ratio of 1.10, −0.1, and 0.45, respectively. The flexural strength of vibration-stirred steel-fiber-reinforced concrete is thus

(10) $$f_{ftm}=1.10f_{tm}{\left(\frac{W}{B}\right)}^{-0.1}\left(1+0.45\lambda_{f}\right)$$

The error range between the split tensile strength test and the calculated value is less than 10%, the average value of the ratio is 1.003, and the mean square error is 0.0596.

### 3.5. Relationship between Tension and Compression Ratio

We performed a regression analysis on the data of Table 3 to establish the relationship between splitting tensile strength and cube compressive strength. The results give

(11) $$f_{ft1}=0.19\left(1+0.09l_{f}\right)f_{fcu1}{}^{0.76}$$

(12) $$f_{ft2}=0.19\left(1+0.12l_{f}\right)f_{fcu2}{}^{0.78}$$

where $f_{ft1}$ is the splitting tensile strength for ordinary-mixed concrete (MPa), $f_{ft2}$ is the splitting tensile strength of vibration-stirred concrete (MPa), $f_{fcu1}$ is the cube compressive strength for ordinary-mixed concrete (MPa), and $f_{fcu2}$ is the cube compressive strength for vibration-stirred concrete (MPa).

### 3.6. Microscopic Morphology of Steel-Fiber-Reinforced Concrete

**Figure 14:**
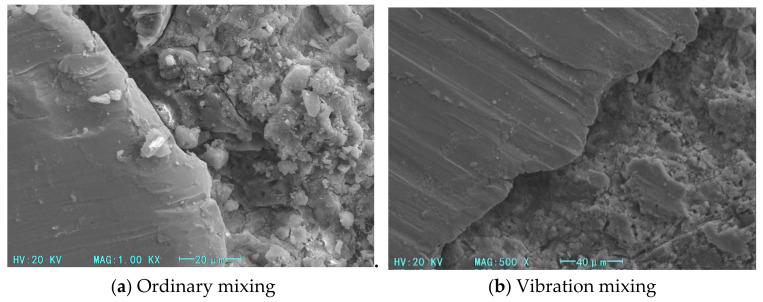
Microstructure of interfacial transition zone. (Magnified 500 times). (**a**) Ordinary mixing (**b**) Vibration mixing.

Figure 14 compares the microstructure of the transition zone of samples of ordinary-mixed and vibration-stirred concrete. The structure of the transition zone between the cement mass and the aggregate body in the ordinary-mixed steel-fiber concrete is loose, with large cracks, large and small pores, and products that are not completely hydrated. The interface transition zone of the steel-fiber-reinforced concrete made by vibration stirring has a compact structure with only a few small cracks and with small pores uniformly distributed. The pores are filled mainly with C–S–H gel, and no obvious flocculent, needle-like, or sheet-like incomplete hydration products appear. The comparison shows that vibration stirring significantly improves the micro-morphology of the interface transition zone, improves the performance of the interface transition zone, reduces the number of cracks and pores, and increases the structural cohesion.

**Figure 15:**
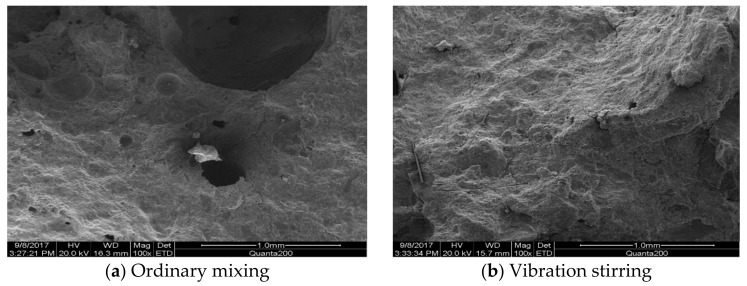
Comparison of morphology of concrete paste with low steel-fiber volume ratio. (Magnified 100 times). (**a**) Ordinary mixing (**b**) Vibration mixing.

**Figure 16:**
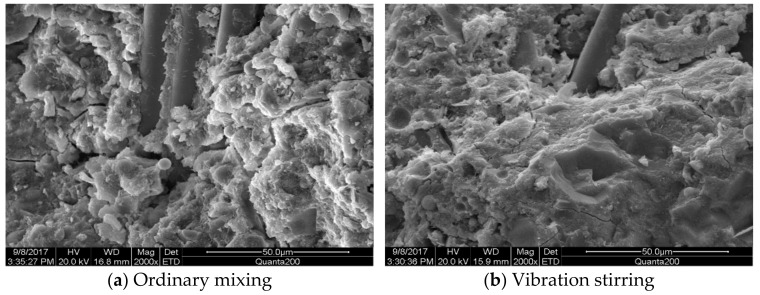
Comparison of morphology of concrete paste with high steel-fiber volume ratio. (Magnified 2000 times). (**a**) Ordinary mixing (**b**) Vibration mixing.

Figure 15 and Figure 16 compare the morphology of ordinary-mixed and vibration-stirred concrete slurry. The morphology of concrete slurry with a low steel-fiber volume ratio was imaged by using electron microscopy. With ordinary mixing, the sample has large pores, a loose structure, and poor adhesion between components. The morphology of concrete slurry with a high steel-fiber volume ratio was also imaged by electron microscopy. For ordinary stirring, the sample has large pores and cracks with the pores dispersed in the mixture. Some steel fibers appear agglomerated and aggregated. For vibration stirring, the hydration product and the aggregate are tightly connected, the steel fibers are evenly wrapped between the mixture, and the pores in the mixture are small and evenly distributed.

## 4. Conclusions

Based on the vibration mixing technology, this paper carried out experimental research on the mechanical properties of steel fiber concrete, and analyzed the microscopic appearance of steel fiber concrete to explore the mechanism of the effect of vibration mixing on the performance of steel fiber. Combining vibration mixing technology, steel fiber volume ratio and matrix strength to study its influence on the performance of steel fiber concrete, it is found that vibration mixing technology can effectively improve the homogeneity and integrity of steel fiber concrete. The main conclusions of this paper are as follows:

1. When the concrete grade is the same, the concrete performance after vibration mixing is higher than that of ordinary mixing. Among them, the improvement efficiency of compressive strength and elastic modulus is more obvious when the amount of steel fiber is low, and the efficiency of splitting tensile strength and flexural strength is more obvious when the amount of steel fiber is high. From the improvement effect of concrete performance and the influence coefficient fitted by the data, it can be seen that the influence of vibration mixing on concrete performance from high to low is: flexural strength > splitting tensile strength > compressive strength > elastic modulus.
2. With different matrix strengths, when the volume ratio of steel fiber is higher than 1%, the higher the matrix strength, the lower the increase in elastic modulus and flexural strength of vibration stirring. The main reason is that when the strength of the matrix increases, the water-binder ratio of the concrete decreases and the amount of cementitious material is large, and the fine cementitious particles cannot be dispersed by vibrating stirring.
3. The addition of steel fiber and vibration mixing can improve the failure morphology of concrete specimens. The larger the volume ratio of steel fiber, the higher the strength of the matrix, the fewer cracks and the less the amount of spalling, and the better the integrity of the specimen.
4. From the study of the microscopic morphology of the vibration mixing steel fiber concrete, it can be seen that the vibration mixing improves the performance of the transition zone of the concrete interface and the slurry, and increases the structural bonding force. Vibration mixing makes the mixtures have a better compactness and a better performance. At the same time, the steel fibers are evenly distributed among the mixtures to avoid agglomeration, which is beneficial to improve the performance of steel fiber concrete.

## Figures and Tables

**Table 1:** Physical properties of coarse aggregate.

Water Absorption	Void Ratio	Crush Index	Apparent Density (kg/m^3^)	Loose Bulk Density (kg/m^3^)	Close Packing Density (kg/m^3^)
0.38%	44.8%	7.6%	2749	1536	1576

**Table 2:** Sieve classification of sand.

Screen Hole Diameter (mm)	Cumulative Sieve Residue
0.15	96.70%
0.3	87.70%
0.6	49.80%
1.18	20.80%
2.36	7.90%
4.75	1.40%

**Table 3:** Mixing proportions of steel-fiber-reinforced concrete.

Matrix Strength	$\rho_{f}\;$	Sand Ratio	Mix Proportion (kg/m^3^)						
			Steel Fiber	Water	Cement	Coarse Aggregate	Fine Aggregate	Fly Ash	Water Reducing Agent
CF40	0	0.37	0	172	300.68	1195.26	701.98	30.07	1.65
	0.5%	0.37	39.25	172	300.68	1173.17	712.06	30.07	1.65
	1.0%	0.37	78.50	172	300.68	1151.09	722.14	30.07	1.65
	1.5%	0.37	117.75	172	300.68	1129.01	732.22	30.07	1.65
	2.0%	0.37	157.00	172	300.68	1106.93	742.31	30.07	1.65
CF50	0	0.36	0	172	347.47	1181.30	664.48	34.75	2.68
	0.5%	0.36	39.25	172	347.47	1159.49	674.29	34.75	2.68
	0.75%	0.36	58.88	172	347.47	1148.58	679.20	34.75	2.68
	1.0%	0.36	78.50	172	347.47	1137.68	684.10	34.75	2.68
	1.5%	0.36	117.75	172	347.47	1116.87	693.91	34.75	2.68
	2.0%	0.36	157.00	172	347.47	1094.06	703.72	34.75	2.68
CF60	0	0.36	0	164	451.79	1078.20	660.83	45.18	4.97
	0.5%	0.36	39.25	164	451.79	1056.84	671.19	45.18	4.97
	1.0%	0.36	78.50	164	451.79	1033.49	681.54	45.18	4.97
	1.5%	0.36	117.75	164	451.79	1011.13	691.90	45.18	4.97
	2.0%	0.36	157.00	164	451.79	989.78	702.25	45.18	4.97

**Table 4:** Main performance parameters of vibration mixer.

Volume (L)	Size (mm^3^)	Mixing Power (kW)	Vibration Power (kW)	Vibration Intensity (G)
60	1900 × 1195 × 1620	2.2	4	1.75

**Table 5:** Experimental results. OM (VM) refers to ordinary (vibration) mixing.

No.	Cube Compressive Strength (MPa)		Axial Compressive Strength (MPa)		Elastic Modulus (GPa)		Tensile Strength (MPa)		Flexural Strength (MPa)		Tensile to Compression Ratio	
	OM	VM	OM	VM	OM	VM	OM	VM	OM	VM	OM	VM
CF40·0	45.89	49.51	33.75	36.53	32.54	33.82	3.57	3.83	5.02	5.81	0.078	0.077
CF40·0.5	48.06	53.33	36.14	39.13	33.76	34.72	3.78	4.09	5.34	6.22	0.079	0.077
CF40·1.0	50.77	54.31	37.06	39.42	34.01	34.95	3.80	4.21	6.01	7.34	0.075	0.078
CF40·1.5	51.62	55.16	37.42	39.61	33.94	34.83	3.99	4.53	7.28	8.13	0.077	0.082
CF40·2.0	53.89	56.49	38.15	40.01	34.21	35.16	4.26	4.94	8.93	9.86	0.079	0.087
CF50·0	52.64	57.13	39.46	42.90	33.79	35.58	3.76	4.07	5.35	6.28	0.071	0.071
CF50·0.5	56.67	63.44	42.76	46.89	35.06	36.41	4.25	4.65	5.51	6.59	0.075	0.073
CF50·1.0	60.24	64.58	44.75	47.54	35.81	36.63	4.39	4.92	6.91	7.96	0.073	0.076
CF50·1.5	62.56	66.86	45.50	48.13	35.74	36.52	4.70	5.39	8.48	9.21	0.075	0.081
CF50·2.0	63.98	67.41	47.01	49.12	36.23	36.94	5.06	5.94	9.67	10.51	0.079	0.088
CF60·0	59.84	64.74	43.25	47.36	35.46	37.57	4.14	4.47	5.92	7.15	0.069	0.069
CF60·0.5	64.67	74.33	47.11	52.23	36.74	38.23	5.09	5.64	6.34	7.86	0.079	0.076
CF60·1.0	72.60	79.14	52.45	56.17	37.91	38.71	5.12	5.75	7.53	8.79	0.071	0.073
CF60·1.5	75.32	81.62	54.32	57.86	37.76	38.57	5.32	6.19	9.96	10.85	0.071	0.076
CF60·2.0	76.39	82.21	55.13	58.27	38.57	39.27	5.57	6.79	11.45	12.40	0.073	0.083

**Table 6:** Experimental and theoretical results for cube compressive strength.

Matrix Strength	Steel-Fiber Volume Ratio (%)	Cube Compressive Strength (MPa)		Ratio
		Expt.	Theory	
CF40	0	49.51	51.93	0.95
	0.50%	53.33	53.76	0.99
	1.00%	54.31	55.59	0.98
	1.50%	55.16	57.41	0.96
	2.00%	56.49	59.24	0.95
CF50	0	57.13	52.69	1.08
	0.50%	63.44	62.56	1.01
	0.75%	63.77	63.63	1.00
	1.00%	64.58	64.69	1.00
	1.50%	66.86	66.82	1.00
	2.00%	67.41	68.95	0.98
CF60	0	64.74	62.34	1.04
	0.50%	74.33	73.36	1.01
	1.00%	79.14	75.86	1.04
	1.50%	81.62	78.35	1.04
	2.00%	82.21	80.85	1.02

## Data Availability

The data presented in this study are available on request from the corresponding author.
